# Clinical implications of single cell sequencing for bladder cancer

**DOI:** 10.32604/or.2024.045442

**Published:** 2024-03-20

**Authors:** REZA YADOLLAHVANDMIANDOAB, MEHRSA JALALIZADEH, FRANCIELE APARECIDA VECHIA DIONATO, KEINI BUOSI, PATRÍCIA A. F. LEME, LUCIANA S. B. DAL COL, CRISTIANE F. GIACOMELLI, ALEX DIAS ASSIS, NASIM BASHIRICHELKASARI, LEONARDO OLIVEIRA REIS

**Affiliations:** 1 UroScience, School of Medical Sciences, University of Campinas, UNICAMP, Campinas, Sao Paulo, 13083-872, Brazil; 2 ImmunOncology, Pontifical Catholic University of Campinas, PUC-Campinas, Campinas, Sao Paulo, 13087-571, Brazil

**Keywords:** Bladder cancer, Urothelial carcinoma, Transitional cell carcinoma, Single-cell sequencing, Tumor heterogeneity, Immunotherapy, Scoping review, Tumor microenvironment

## Abstract

Bladder cancer (BC) is the 10th most common cancer worldwide, with about 0.5 million reported new cases and about 0.2 million deaths per year. In this scoping review, we summarize the current evidence regarding the clinical implications of single-cell sequencing for bladder cancer based on PRISMA guidelines. We searched PubMed, CENTRAL, Embase, and supplemented with manual searches through the Scopus, and Web of Science for published studies until February 2023. We included original studies that used at least one single-cell technology to study bladder cancer. Forty-one publications were included in the review. Twenty-nine studies showed that this technology can identify cell subtypes in the tumor microenvironment that may predict prognosis or response to immune checkpoint inhibition therapy. Two studies were able to diagnose BC by identifying neoplastic cells through single-cell sequencing urine samples. The remaining studies were mainly a preclinical exploration of tumor microenvironment at single cell level. Single-cell sequencing technology can discriminate heterogeneity in bladder tumor cells and determine the key molecular properties that can lead to the discovery of novel perspectives on cancer management. This nascent tool can advance the early diagnosis, prognosis judgment, and targeted therapy of bladder cancer.

## Introduction

Single-cell sequencing technologies were pioneered in the early 90s and gained widespread attention in 2013 when they were named “Nature Methods”. These technologies are superior to traditional bulk sequencing methods due to their ability to map heterogenicity among individual cells of the same tissue. In short, these technologies isolate cells before administrating highly sensitive next-generation sequencing (NGS), which can sequence the very small amount of material available at the single-cell level. There are numerous techniques for isolating cells. The simplest method is separating cells one by one by micropipette under a microscope. The limiting dilution technique involves separating and suspending the cells in liquid before distributing the suspension in multi-well plates at the concentration of 0.5 cells per well. Microfluidic separation is a more advanced high throughput technique with relatively low cost. Single-nucleus separating is sometimes used instead of whole-cell separating to increase the durability of samples [1,2].

RNA is the most commonly sequenced molecule in these technologies (RNAseq), allowing researchers to map gene activity at the time of biopsy in each cell (transcriptional variability). In the field of oncology, this allows for the assessment of the tumor microenvironment (TME) at the single-cell level. Whole-exome sequencing (WES) maps the functional DNA and whole-genome sequencing (WGS) maps the entire DNA. Targeted NGS (tNGS) maps a specific panel of related genes that may be altered in single cells. T cell receptor sequencing (TCR-seq) maps the TCR-related RNA activity of single T cells [2–4]. Recently, NGS has been combined with the detection of other molecules such as proteins to concomitantly map them on individual cells (multi-omics). Chromatin immunoprecipitation (CHIP-seq), REAP-seq (RNA and surface protein), CITE-seq, and QuRIE-seq (RNA and epitopes) are among these methods [5–8].

Bladder cancer (BC) accounts for 3% of all new cancer diagnoses with 0.5 million new cases and 0.2 million deaths per year [9]. It is the 13th leading cause of cancer-related death in the world [10]. The 5-year overall survival rate for BC can be as high as 77% in non-metastatic disease and only 6% in metastatic BC. This cancer is four times more common in men (9.6 per 100,000) than in women (2.4 per 100,000). Tobacco smoking, positive family history, and exposure to chemicals are the main risk factors for this malignancy [11]. The frequency of BC rises with age; more than 90% of diagnoses occur in patients over 55 years of age [11]. Bladder cancer is classified into muscle-invasive bladder cancer (MIBC) and non-muscle-invasive bladder cancer (NMIBC) [9,12]. In addition to surgery, patients may receive radiotherapy, chemotherapy with mitomycin C, epirubicin, thiotepa, gemcitabine, platinum-based agents, doxorubicin, Bacillus Calmette Guerin (BCG) immunotherapy, and Immune Checkpoint Inhibitor (ICI) THERAPY [13]. Despite advances in available therapeutic methods, the clinical outcome of patients with bladder cancer remains unsatisfactory.

In this review, we summarize the clinical implications of single-cell sequencing technologies in bladder cancer.

## Methods

The design and implementation of this scoping review were guided by Arksey et al.’s [14] methodological framework, described by the Joanna Briggs Institute [15]. To organize the information, the PRISMA model [16] was used and we followed the PRISMA-ScR recommendations [17].

### Inclusion and exclusion criteria

This review focuses on the clinically significant application of single-cell sequencing technologies in bladder cancer. Inclusion criteria are *in vitro* or *in vivo* studies involving single-cell sequencing in bladder cancer published in any language. Exclusion criteria are letters, reviews, conference abstracts, editorials, unrelated articles, replies, unpublished articles, and articles without full-text access.

### Information sources

A comprehensive search of the literature published until February 2023 was conducted using Emtree language and medical subject headings (MeSh), in Pubmed, Embase, and the Cochrane Central Register of Controlled Trials (CENTRAL) databases. A broad search strategy was performed using a combination of the following search terms: ((urothelial cancer OR bladder cancer) AND single-cell sequencing). We supplemented this search with manual searches of the relevant articles identified through the Scopus, and Web of Science. Details of the search strategy are available in Appendix 1.

### Data extraction

We used the Rayyan platform to screen articles and extract data. The software automatically removed duplicates; the remaining exclusions were manually performed by two researchers. Titles and abstracts were separately screened by the first and third authors with a portion of articles double screened for application of inclusion and exclusion criteria. The second author separately screened all articles. Data were extracted by the first three authors.

## Results and Discussion

**Figure 1:**
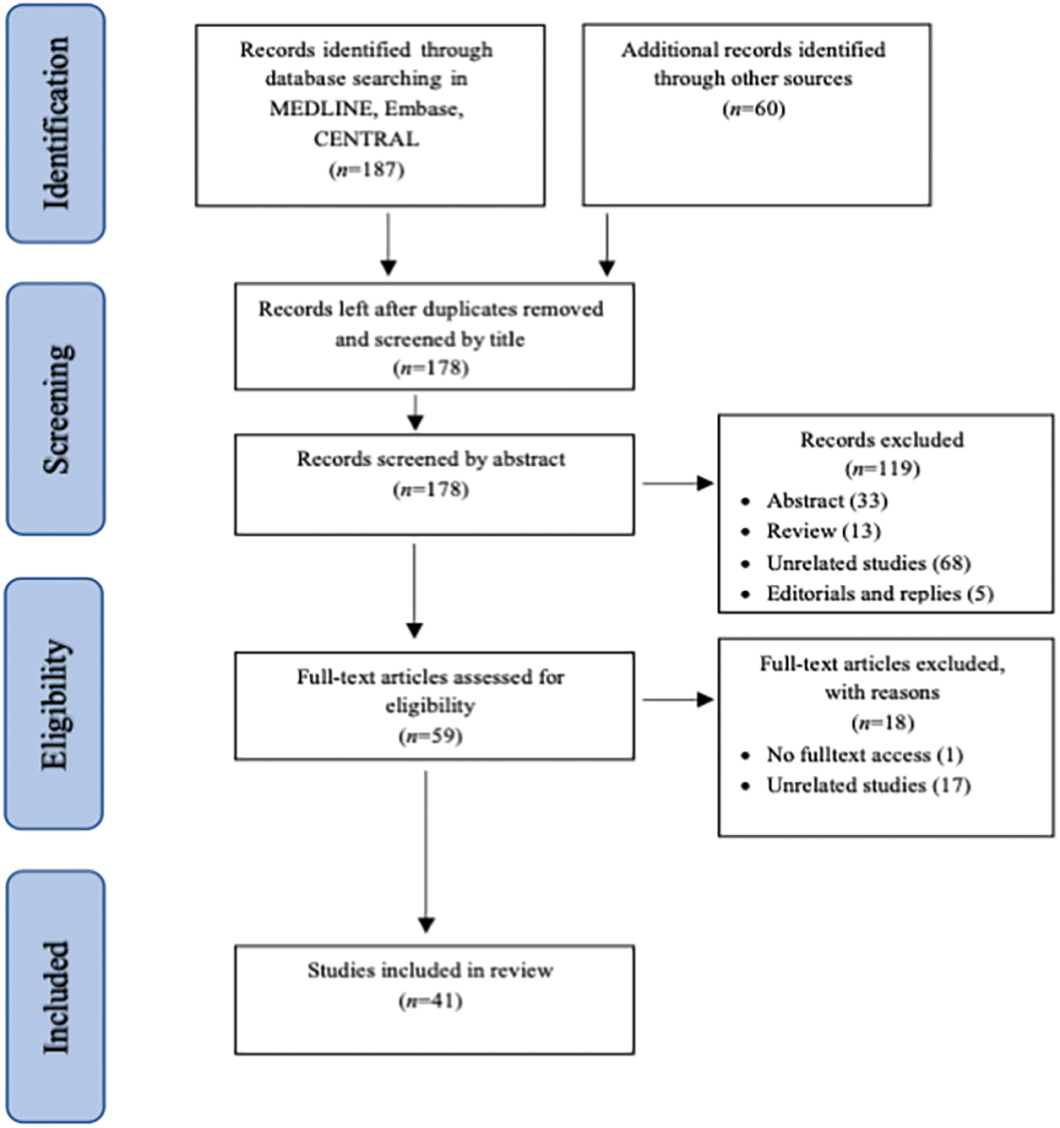
Process of identification and inclusion of studies—PRISMA flow diagram.

Our initial search resulted in 247 citations (187 citations from electronic databases and 60 citations from other sources). After removing the duplicates, the first two authors independently reviewed the remaining 180 titles and extracted those that clearly did not meet the inclusion criteria. The first three authors independently reviewed the abstracts of 135 potentially eligible studies. The full text of the remaining 66 studies was obtained and evaluated in accordance with the inclusion criteria. Any disagreement over inclusion was resolved by discussion. Finally, 41 studies were included in the review (Fig. 1).

Table 1 summarizes the included articles. The first 19 articles performed single-cell sequencing on samples acquired from patient tumors [18–36]. Five studies performed this technique *in vitro* or on animal BC models [37–41]. The remaining used public database [42–58]. All studies classified single cells and defined cell clusters contributing to the TME of BC. They identified clinically important cell subtypes by analyzing patient outcomes or responses to treatment.

**Table 1:** The summary of the included articles

	Main study	Studied cells/Tissue	Clinical implication	Key findings	Validation
[18] Gouin 3rd et al.	snRNA seq, spatial transcriptomics, and sc resolution spatial proteomic of patient tumors	High grade urothelial MIBC	Response prediction	An epithelial cell subtype with overexpression of Cadherin 12 can predict response to surgery and ICI	Validated by analyzing TCGA and IMvigor databases
[19] Ma et al.	scRNA seq of patient tumors	CAFs	Response and outcome prediction	A subpopulation of CAF with overexpression of urea transporter SLC14A1 can confer to BC stemness	Validated by comparing to a previously published study
[20] Lindskrog et al.	snRNA seq and spatial transcriptomic of patient tumors	NMIBC	Outcome prediction	Molecular classification of tumors based on bulk RNA seq has prognostic value	Validated by bulk seq and spatial transcriptomics
[21] Fehlings et al.	Bulk RNA seq, TCR seq, and CITE-seq of patient blood	PBMCs	Response prediction	Subtypes of T cells are associated with response to ICI therapy	Validated by analyzing IMvigor database
[22] Wu et al.	scRNA seq of patient tumors	Different BC tumors and NAT	Potential therapeutic target	Treg subtype that highly expresses TIGIT and IL-32 has immunosuppressive properties, targeting TIGIT can suppress metastasis	Validated by analyzing TCGA and GTEx public databases
[23] Xu et al.	IHC, scRNA seq and flow cytometry of patient tumors	TAMs in MIBC	Outcome prediction	IL-10+ TAMs are associated with immune evasive tumor environment	Validated by analyzing TCGA public database
[24] Chen et al.	scRNA seq of patient tumors	BC and NAT	Outcome prediction	iCAFs may have prognostic value, tumor cells downregulated MHC-II, identified immune cell subgroups in the TME	Validated by analyzing TCGA public database
[25] Oh et al.	scRNA seq and TCR seq of patient tumors	T cells from BC and NAT	Response prediction	Subtypes of cytotoxic CD4+ T cells can predict response to ICI	Validated by analyzing IMvigor database
[26] Liang et al.	scRNA seq of patient tumors	UTUC	Response and outcome prediction	A gene expression score describing macrophage exhaustion can predict response to ICI and has prognostic value	Compared results to other cohorts involving bulk RNA seq of urothelial BC and UTUC
[27] Tao et al.	Bulk seq and cytometry of patient tumors	MIBC	Outcome prediction	A TP53-related score can predict BC outcome	Validated by analyzing GEO public database
[28] You et al.	Bulk RNA seq of patient tumors	NMIBC and NAT	Outcome prediction	Expression level of cell cycle regulating gene SKA3 can predict NMIBC progression	Validated by analyzing TCGA and GEO public databases
[29] Wang et al.	scRNA seq and ATAC-seq of patient tumors	Different BC subtypes	Outcome prediction	Elevated expression of EZH2 gene predicts recurrence, clustered heterogenous cells of TME focusing on stem cells and EMT	
[30] Lai et al.	scRNA seq of patient tumors	Different BC subtypes		Clustered heterogenous cells of TME, investigated EMT, matched their findings with histopathology	
[31] Li et al.	scExome seq of one patient tumor	MIBC and NAT		Found single ancestral cells for all tumor cells, identified mutated genes	Compared to bulk exome seq of a previously published cohort study
[32] Banchereau et al.	scRNA seq and TCR seq of IMvigor patients	Metastatic urothelial carcinoma and lung cancer	Response prediction	A subtype of tissue-resident memory T cells (CD103+CD8+) predicts response to ICI	Validated by analyzing GEO publicly available database
[33] Yang et al.	sc-DNA seq of patient tumors	Stem cells and non-stem cells in BC		Identified mutated genes associated with self-renewal of stem cells, identified two phylogenic origins of stem cells in BC	
[34] Luo et al.	scRNA seq of patient tumors	Different BC subtypes		Clustered heterogenous cells of TME, matched the scRNA-seq findings with the histopathology of each tumor	
[35] Warrick et al.	scRNA seq of one patient tumor	Squamous differentiated urothelial carcinoma	Response prediction	Found single ancestral cells for all tumor cells, found expression signature for squamous differentiated cells that may predict response to platinum-based chemotherapy	
[36] Zhang et al	scRNA seq of one patient tumor	Squamous cell carcinoma of bladder and NAT		Identified gene expression differences between cancer cells and NAT	
[37] Tanaka et al.	scRNA seq of cell culture	5637 human urothelial carcinoma	Response prediction	Gene COX7B and its surrogate marker CD63 predict response to platinum-based medication	Validated by analyzing TCGA public database and inhouse IHC of patient BC tumors
[38] Sfakianos et al.	scRNA seq of human and murine BC models	BBN induced murine BC plus xenografts of patient MIBCs in nude mouse		Clustered heterogenous cells of TME and identified lineage plasticity origins leading to TME heterogeneity	
[39] Kerzeli et al.	scRNA seq of mouse BC	BBN induced murine BC	Potential new treatment	Clustered heterogenous cells of TME and introduced CpG-oligodeoxynucleotides as a potential treatment	
[40] Esteso et al.	scRNA seq and flow cytometry on lymphocytes	PBMCs		PBMCs exposed to BCG show expansion of IFN-γ producing γδ T-cells with high anti-neoplastic functions	
[41] Podojil et al.	scRNA seq and mass cytometry of mouse tumors	BBN induced murine BC	Potential therapeutic target	B7-H4, a protein with T cell inhibition properties expressed by luminal tumors can be targeted by immunotherapy	Validated by expression analysis of patient tumors
[42] Zhou et al.	Analyzed TCGA public database	B cells from BC	Response and outcome prediction	B cell senescence-related gene signature can predict response to ICI and has prognosis value	Validated by analyzing GEO and IMvigor databases plus inhouse RT-qPCR and IHC
[43] Liu et al.	Analyzed TCGA public database plus scRNA seq patient tumors	BC and NAT	Response and outcome prediction	CD39 gene has prognostic value, inhibiting CD39 increases intratumor natural killer cell invasion and enhances response to cisplatin but not ICI	Validated by inhibiting CD39 in different murine models
[44] Chen et al.	Analyzed GEO public database	Human BC	Response and outcome prediction	Markers of iCAF can predict variation in immunological TME, prognosis, and response to ICI, an iCAF subtype can inhibit proliferation and migration of carcinoma cells *in vitro*	Validated by analyzing TCGA, GEO, and IMvigor databases Validated the iCAF subtype properties by co-culturing them with T24 cell lines
[45] Zhao et al.	Analyzed GEO, ENA, and TCGA public database	Epithelial cells in NMIBC, MIBC, and NAT	Outcome prediction	Expression level of SLC16A3 gene has prognostic value	Validated by inhouse IHC for MCT4 protein in patient tumors
[46] Du et al.	Analyzed TCGA public database	BC and NAT	Response and outcome prediction	Calumenin gene is correlated to epithelial-mesenchymal transition, ferroptosis, TP53 mutation, and may predict response to ICI	Validated by inhouse qPCR and IHC of patient tumors
[47] Chen et al.	Analyzed GEO and TCGA public databases	Pan-cancer	Response and outcome prediction	JunB proto-oncogene can be the hub gene responsible for immunosuppressive TME and may predict response to ICI	Validated by various inhouse experiments on patient BC tumors and T24 urothelial carcinoma cell lines
[48] Xiang et al.	Analyzed TCGA public database	BC and NAT	Outcome prediction	Ferroptosis-associated lncRNAs are specifically expressed in TME and have prognostic value	Validated by inhouse scRNA seq on patient tumors
[49] Wang et al.	Analyzed IMvigor 210 and CheckMate275 public databases	Metastatic urothelial carcinoma	Response and outcome prediction	A “protumorigenic inflammation signature” can predict response to ICI and has prognostic value, the cell subtype associated with the signature are myeloid phagocytic cells	Validated by inhouse scRNA seq on patient tumors
[50] Li et al.	Analyzed GEO, ArrayExpress, TCGA, and GTEx public databases	BC and NAT	Outcome prediction	Enhanced immune cell infiltration in tumors with high expression level of PTTG1	Validated by inhouse microarray and IHC of patient tumors
[51] Jiang et al.	Analyzed TCGA public database	BC and NAT	Outcome prediction	Expression level of TAM-related genes has prognostic value	Validated by analyzing GEO public database
[52] Yao et al.	Analyzed GEO public database	MIBC cells with stemness properties	Outcome prediction	The gene DBI is associated with stemness and its expression is associated with higher grade tumors, acetaminophen can reduce DBI expression	Validated by analyzing TCGA public database
[53] Yu et al.	Analyzed GEO and TCGA public databases	scRNA seq of normal bladder was used to deconvolute MIBC data	Outcome prediction	Identifying cell subtypes has prognostic value	Validated by analyzing TCGA, GEO, and MSKCC public databases
[54] Acharjee et al.	Analyzed TCGA public database	BC and colon cancer	Diagnosis and outcome prediction	LGALS4 (Galectin-4) gene can be a prognostic and diagnostic marker for BC	Validated by analyzing GEO public database
[55] Das et al.	Analyzed TCGA and GEO public databases	Pan-cancer	Response and outcome prediction	A “Cancer-Specific Immune Prognostic Signature” can predict response to ICI	Validated BC response to ICI by analyzing IMvigor public database
[56] Hu et al.	Analyzed TCGA, SRA, GEO, ArrayExpress, and IMvigor public databases	BC	Response and outcome prediction	TME of different tumors can be classified into four subtypes to predict response to ICI and prognosis	
[57] Wang et al.	scRNA seq of patient urine	Exfoliated cells in urine	Diagnosis	Hexokinase 2 can be a urine marker for BC	
[58] Chen et al.	Microfluidic immunoassay of patient urine	Exfoliated cells in urine	Diagnosis	Immunofluorescent staining for oncoproteins CK20 and CD44v6 can have diagnostic value	Validated by sc-whole genome seq

Note: All *patient tumors* are bladder cancer samples from human patients. AIM2, absent in melanoma 2; ATAC-seq, assay for transposase-accessible chromatin using sequencing; BBN, N-butyl-N-(4-hydroxybutyl)-nitrosamine; BC, bladder cancer; DBI, diazepam binding inhibitor; EMT, epithelial-mesenchymal transition; ENA, European nucleotide archive (www.ebi.ac.uk/ena); EZH2, enhancer of zeste 2 polycomb repressive complex 2 Subunit; IL, interleukin; IHC, immunohistochemistry; lncRNAs, long non-coding RNAs; MIBC, muscle invasive bladder cancer; MCT4, monocarboxylate transporter 4 protein coded by SLC16A3 gene; MSKCC, memorial sloan-kettering cancer center; NAT, normal adjacent tissue; PTTG1, pituitary tumor transforming gene 1; PBMC, peripheral blood mononuclear cells; sc, single cell; sn, single nucleus; seq, sequencing; ICI, immune checkpoint inhibition; IMvigor, lung and bladder cancer clinical trials of atezolizumab; ICI (anti-programmed cell death ligand 1); Treg, T regulatory; TIGIT, T-cell immunoreceptor with immunoglobulin and immunoreceptor tyrosine-based inhibitory motif domain; CAF, cancer associated fibroblast; iCAF, inflammatory cancer-associated fibroblasts; SRA, sequence read archive (www.ncbi.nlm.nih.gov/sra); TME, tumor microenvironment; TAM, tumor-associated macrophage; RT-qPCR, real-time quantitative PCR; UTUC, upper tract urothelial carcinoma.

Public databases: 29 articles analyzed public cancer databases as part of their study. Gene Expression Omnibus (GEO) [59] is currently the largest database containing single-cell sequencing data of cancer patients. Other databases such as The Cancer Genome Atlas project (TCGA) [60], ArrayExpress [61], and Genotype Tissue Expression project (GTEx) [62] mainly contain bulk sequencing data. TISCH [63] and The Cancer Single-Cell State Atlas (CancerSEA) [64] are lesser-known public databases of single-cell RNA sequences focusing on TME. The website of CellPress provides a review of highlighted reveals by these pooled public databases [65].

Fourteen studies analyzed these publicly available databases to generate a hypothesis [42–56]. Nine studies validated their hypothesis by in-house experiments [42–50]. Five studies validated their hypothesis by analyzing other databases [51–55].

Bladder cancer tends to be a heterogeneous tumor [66]. Nine studies investigated this heterogeneity by single-cell analysis of BC tumors and normal adjacent tissue (NAT). They classified cells into clusters and showed their ancestral origin [31–36,38,39,56].

The clinical implication of single-cell sequencing is evident in seven studies [18,19,25,32,42,55,56]. These studies show that separating cells of a patient’s tumor and characterizing cell subtypes can improve clinical decision-making by predicting the tumor’s response to different treatments.

### Limitations of studies

Current single-cell sequencing techniques are extremely complex and involve multiple steps. The process of separating cells could damage the cell contents. Two studies addressed this issue by performing single-nucleus separation instead of whole-cell separation; the nucleus is less prone to degradation during the separation procedure [18,20].

Analyzing the high-dimensional data generated by single-cell sequencing is extremely complex and suffers from “the curse of dimensionality” [67], i.e., too much information [2,3,68–70]. Most studies addressed this issue by filtering for genes of interest as the first step of their analysis. For example, Zhou et al. [42] gathered a list of senescence-related genes from the website CellAge (http://genomics.senescence.info/cells) and only investigated related data in the TCGA database to generate their B cell senescence-related gene signature (Table 1). Other studies addressed this complexity through the reduction of dimensions (excluding insignificant genes). The most common dimension reduction techniques were clustering using the R package Seurat and visualizing using t-SNE [71].

Public databases such as TCGA and GEO pool data from various sequencing studies without providing sufficient data on heterogeneity among their techniques and patient factors. This is less important when sequencing DNA since DNA is a stable molecule designed by nature to maintain its integrity throughout an animal’s life. However, RNA sequencing is tricky since RNA is a transient molecule, only expressed when the cell requires it and degraded fairly rapidly. At room temperature, mRNA has a half-life of 16 h [72]. It is assumed that all RNA sequencing data pooled in these databases are acquired from analyzing fresh samples using similar techniques. However, a sentence in the article by Banchereau et al. (Table 1) casts doubt on this assumption: “…a bulk RNAseq analysis was performed on formalin-fixed paraffin-embedded tumor tissue”. Banchereau et al. experiments are a major contributor to IMvigor studies used by many articles included in this review [32].

It is important to keep in mind that transcription in cells is highly dependent on environmental factors. Temperature, oxygen level, stress, and even the circadian rhythm (time of the day) can influence transcription. Many commonly used medications such as steroids directly affect transcription [73–76]. As Yao et al. showed (Table 1), the over-the-counter drug acetaminophen influences the expression level of the gene, they found to be associated with BC tumor grade [52].

## Conclusion

Single-cell analysis is a relatively new tool that aids in understanding the complexity of bladder cancer tumor’s microenvironment. Included studies in our scoping review collectively show that identifying certain cell subtypes in these heterogenous tumors may help clinicians in predicting response to expensive or invasive therapies such as immune checkpoint inhibitors or surgery with or without neoadjuvant chemotherapy. However, there are currently no clinical trials showing the benefit of performing this test on the overall well-being of the patients. Future single-cell sequencing studies should include epidemiological data, environmental factors, as well as comprehensive clinical data to improve our understanding of bladder cancer etiology and its heterogenous response to treatment.

## Supplementary Materials



## Data Availability

Not applicable.

## Abbreviations

BC: Bladder cancer
CAF: Cancer associated fibroblast
ICI: Immune checkpoint inhibitor
scRNA seq: Single cell RNA sequencing
snRNA seq: Single nucleus rna sequencing
TAM: Tumor associated macrophages
TME: Tumor microenvironment

## Supplementary Materials

The supplementary material is available online at https://doi.org/10.32604/or.2024.045442.
