# Supplementary material for: Clinical implications of single cell sequencing for bladder cancer
Source: Oncol Res. 2024 Mar 20;32(4):597–605. doi: 10.32604/or.2024.045442 (PMC10972735; doi:10.32604/or.2024.045442)
Supplement: Supplementary file 1 [file OncolRes-32-45442-s001.docx]

**Appendix 1. Search strategies**

**Pubmed**

"Single-Cell RNA-Seq" OR "Single-Cell RNA-Seq" OR "RNA-Seq, Single-Cell" OR "Single Cell RNA Seq" OR "Single Cell RNA Seq" OR "ScRNA-seq" OR "ScRNA-seq" OR "ScRNA-seq" OR "ScRNA seq" OR "Sc-RNAseq" OR "Sc RNAseq" OR "ScRNAseq" OR "Single-Cell RNA Seq" OR "RNA Seq, Single-Cell" OR "Seq, Single-Cell RNA" OR "Seqs, Single-Cell RNA" OR "Single-Cell RNAseq" OR "RNAseq, Single-Cell" OR "Single Cell RNAseq" OR "scATAC-seq" OR "scATACseq" OR "single-cell ATAC sequencing" OR "scWGS-seq" OR "single-cell whole-genome sequencing" OR "scWGSseq" OR "scWES-seq" OR "single-cell whole-exome sequencing" OR "scDNA-seq" OR "scDNAseq" OR "Single Cell DNAseq" OR "Single-Cell DNAseq" OR "Single-Cell DNA sequencing" OR "Single Cell DNA sequencing" AND "Neoplasm, Urinary Bladder" OR "Urinary Bladder Neoplasm" OR "Bladder Tumors" OR "Bladder Tumor" OR "Tumor, Bladder" OR " Bladder Carcinoma" OR "Tumors, Bladder" OR "Neoplasms, Bladder" OR "Bladder Neoplasms" OR "Bladder Neoplasm" OR "Neoplasm, Bladder" OR "Urinary Bladder Cancer" OR "Cancer, Urinary Bladder" OR "Malignant Tumor of Urinary Bladder" OR "Cancer of the Bladder" OR "Bladder Cancer" OR "Bladder Cancers" OR "Cancer, Bladder" OR "Cancer of Bladder" OR "Urothelial Cancer" OR "Urothelial Carcinoma"

**Central (ovid)**

("Single-Cell RNA-Seq" OR "Single-Cell RNA-Seq" OR "RNA-Seq, Single-Cell" OR "Single Cell RNA Seq" OR "Single Cell RNA Seq" OR "ScRNA-seq" OR "ScRNA-seq" OR "ScRNA-seq" OR "ScRNA seq" OR "Sc-RNAseq" OR "Sc RNAseq" OR "ScRNAseq" OR "Single-Cell RNA Seq" OR "RNA Seq, Single-Cell" OR "Seq, Single-Cell RNA" OR "Seqs, Single-Cell RNA" OR "Single-Cell RNAseq" OR "RNAseq, Single-Cell" OR "Single Cell RNAseq" OR "scATAC-seq" OR "scATACseq" OR "single-cell ATAC sequencing" OR "scWGS-seq" OR "single-cell whole-genome sequencing" OR "scWGSseq" OR "scWES-seq" OR "single-cell whole-exome sequencing" OR "scDNA-seq" OR "scDNAseq" OR "Single Cell DNAseq" OR "Single-Cell DNAseq" OR "Single-Cell DNA sequencing" OR "Single Cell DNA sequencing"):ti,ab,kw (Word variations have been searched)

("Neoplasm, Urinary Bladder" OR "Urinary Bladder Neoplasm" OR "Bladder Tumors" OR "Bladder Tumor" OR "Tumor, Bladder" OR " Bladder Carcinoma" OR "Tumors, Bladder" OR "Neoplasms, Bladder" OR "Bladder Neoplasms" OR "Bladder Neoplasm" OR "Neoplasm, Bladder" OR "Urinary Bladder Cancer" OR "Cancer, Urinary Bladder" OR "Malignant Tumor of Urinary Bladder" OR "Cancer of the Bladder" OR "Bladder Cancer" OR "Bladder Cancers" OR "Cancer, Bladder" OR "Cancer of Bladder" OR "Urothelial Cancer" OR "Urothelial Carcinoma"):ti,ab,kw (Word variations have been searched)

**Embase**

('bladder cancer'/syn OR 'transitional cell carcinoma'/exp OR 'transitional cell carcinoma of the bladder'/syn) AND ('single cell sequencing'/syn OR 'single cell rna seq'/syn OR 'single cell dna sequencing'/syn OR 'single-cell whole-genome sequencing' OR 'single-cell atac sequencing' OR 'single-cell whole-exome sequencing')

**Scopus**

[( TITLE-ABS ( "Neoplasm, Urinary Bladder"  OR  "Urinary Bladder Neoplasm"  OR  "Bladder Tumors"  OR  "Bladder Tumor"  OR  "Tumor, Bladder"  OR  " Bladder Carcinoma"  OR  "Tumors, Bladder"  OR  "Neoplasms, Bladder"  OR  "Bladder Neoplasms"  OR  "Bladder Neoplasm"  OR  "Neoplasm, Bladder"  OR  "Urinary Bladder Cancer"  OR  "Cancer, Urinary Bladder"  OR  "malignant tumor off urinary bladder"  OR  "cancer off the bladder"  OR  "Bladder Cancer"  OR  "Bladder Cancers"  OR  "Cancer, Bladder"  OR  "cancer off bladder"  OR  "Urothelial Cancer"  OR  "Urothelial Carcinoma" ) )  AND  ( TITLE-ABS ( "Single-Cell RNA-Seq"  OR  "Single-Cell RNA-Seq"  OR  "RNA-Seq, Single-Cell"  OR  "Single Cell RNA Seq"  OR  "Single Cell RNA Seq"  OR  "secrna-seq"  OR  "secrna-seq"  OR  "secrna-seq"  OR  "secrna seq"  OR  "sec-rnaseq"  OR  "sec rnaseq"  OR  "secrnaseq"  OR  "Single-Cell RNA Seq"  OR  "RNA Seq, Single-Cell"  OR  "Seq, Single-Cell RNA"  OR  "sets, single-cell rna"  OR  "single-cell rnase"  OR  "rnase, single-cell"  OR  "single cell rnase"  OR  "secatac-seq"  OR  "secatacseq"  OR  "single-cell ATAC sequencing"  OR  "secwgs-seq"  OR  "single-cell whole-genome sequencing"  OR  "secwgsseq"  OR  "secwes-seq"  OR  "single-cell whole-exom sequencing"  OR  "secdna-seq"  OR  "secdnaseq"  OR  "single cell dnase"  OR  "single-cell dnase"  OR  "Single-Cell DNA sequencing"  OR  "Single Cell DNA sequencing" ) )](https://www.scopus.com/results/documentSpellSuggest.uri?sort=plf-f&src=s&mltEid=&mltAll=t&sid=1ab46c3f0e12fd21274bb1de57278ba5&sot=comb&sdt=comb&sessionSearchId=1ab46c3f0e12fd21274bb1de57278ba5&origin=resultslist)
